# A Vision-Based Approach for the Analysis of Core Characteristics of Volcanic Ash

**DOI:** 10.3390/s21217180

**Published:** 2021-10-29

**Authors:** Bruno Andò, Salvatore Baglio, Salvatore Castorina, Vincenzo Marletta

**Affiliations:** Department of Electric Electronic and Information Engineering (DIEEI), University of Catania, 95124 Catania, Italy; salvatore.baglio@unict.it (S.B.); salvatore.castorina@unict.it (S.C.); vincenzo.marletta@dieei.unict.it (V.M.)

**Keywords:** volcanic ash, ash fall-out, ash granulometry, core-characteristics estimation, vision-based paradigm

## Abstract

Volcanic ash fall-out represents a serious hazard for air and road traffic. The forecasting models used to predict its time–space evolution require information about the core characteristics of volcanic particles, such as their granulometry. Typically, such information is gained by the spot direct observation of the ash collected at the ground or by using expensive instrumentation. In this paper, a vision-based methodology aimed at the estimation of ash granulometry is presented. A dedicated image processing paradigm was developed and implemented in LabVIEW™. The methodology was validated experimentally using digital reference images resembling different operating conditions. The outcome of the assessment procedure was very encouraging, showing an accuracy of the image processing algorithm of 1.76%.

## 1. Introduction

The ash fall-out phenomenon due to the explosive activity of volcanoes represents a considerable risk factor for people’s health and everyday life activities [1]. In particular, common consequences of ash fall-out can be experienced in the areas of road safety, sanitation systems [2], agriculture [3], health [4] and air traffic [5]. As an example, the eruption of the Icelandic Eyafjallajokull volcano in 2010, which is considered to have been one of the most intense eruptions, caused major damages and problems for people and infrastructures.

Moreover, ash eruptions represent a serious hazard for air traffic, including potential damages to aircraft components [5,6], flight safety issues, and inconveniences at airports. In many cases, the consequences of ash fall-out include flight cancellations or temporary closures of airports, with difficulties for passengers and loss of profit for airlines and airport operators. This is the case for the international Fontanarossa airport in Catania, in the south of Italy, close to Mount Etna, the largest active volcano in Europe. As an example, in the last decades during eruptions of Etna, the Fontanarossa airport has been repeatedly declared inappropriate for take-offs and landings due to the ash plumes spewed by the volcano and resulting volcanic ash fall-out, creating great inconvenience to passengers along with financial loss for airlines and airport operators.

In order to promptly activate emergency actions, the availability of reliable monitoring systems designed to detect the intensity and characteristics (e.g., particles dimensions) of the ash fall-out phenomenon would be of strategic importance. The size of volcanic ash particles is commonly expressed in terms of granulometry classes, ranging from 0.1 mm to 4.0 mm. These data can be efficiently used to predict the time–space evolution of volcanic particles by using dedicated models, which use both the meteorological quantities (e.g., wind speed and direction) and the characteristics of ash fall-out (flow and granulometry) [7]. Forecasting models are useful to implement the optimized planning of emergency actions; e.g., to clean roads, to restore the airport functionalities, and to manage the air traffic during the crucial phase of the addressed phenomenon. Such models require the characteristics of the ash fall-out to be known in order to obtain optimal simulation results; however, as shown in [8], this information is not always available with traditional monitoring approaches.

Different techniques have been used worldwide to alert airports of volcanic activity. The real-time monitoring of explosive volcanoes by seismic and infrasonic instruments [9,10,11], forecasts of ash dispersion and deposition [12] and the detection of approaching ash clouds using high-cost instrumentation typically based on satellites [13], X-Band dual-polarization radars [14], ground thermal infrared cameras (TIRs) [15] or ground-based microwave weather radars [16] and vision systems [17,18,19], and laser diffraction and image processing techniques [20] are some examples of adopted solutions. In particular, in [19], a method for characterizing the properties of volcanic ash, both luminance and particle shape, based on an image processing approach is reported. The proposed method is very accurate but requires particle cleaning, drying, and sieving steps, leading to considerable elaboration times. A convolutional neural network (CNN) for the classification of volcanic ash is proposed in [21]. In [22], the authors propose a method to estimate multiple 3D geometric shape descriptors using the X-ray computed tomography method.

Although the above solutions provide accurate information about volcanic activity and the ash fall-out phenomenon, they are expensive, difficult to install and maintain and provide information with a low degree of spatial resolution (typically, they are used to perform spot measurements).

Conversely, to provide a reliable support to authorities managing the different kind of emergencies caused by the ash fall-out, the forecasting models used to predict the time–space evolution of ash dispersion should be supplied by spatially distributed and continuous-time information. In particular, the granulometry and shape of the particles and their sedimentation rate represent mandatory information since these could affect the fall velocity.

In this framework a distributed sensor network of low-cost monitoring stations would represent a suitable solution for performing continuous monitoring and gaining a high spatial resolution awareness of the ash fall-out phenomenon.

Under the SECESTA project [23], an early-warning system (EWS) has been developed, which is based on a distributed network of low-cost multi-sensor nodes for the measurement of quantities characterizing falling-out volcanic particles [24]. By definition, this approach leads to well spatially distributed information at the expense of the high accuracy provided by high-cost instrumentation. However, the latter is mandatory to perform spot measurements in the case of the specific needs evidenced by the EWS. The sensing node developed through the SECESTA project consists of a collector to convey ash into an instrumented tank. An infrared barrier is used for the monitoring of ash levels, which allows the ash flowrate to be estimated indirectly. Moreover, a piezoelectric sensor, placed within the collector, allows for the indirect estimation of ash granulometry. Each monitoring station is also equipped with meteorological sensors to feed models predicting the space–time evolution of the ash fall-out phenomenon.

As a further development, in [25], the authors addressed a novel methodology for ash flow-rate estimation and a strategy for the discrimination of volcanic ash from other types of sediments. In particular, a digital magnetometer is used for the selective discrimination of ash particles (which are paramagnetic).

The main limitation of the approach proposed in [24,25] to measure ash granulometry is related to the failures caused by multiple bounces from the undefined particle-side hitting the piezoelectric sensor and/or particle agglomerates, which could seriously impact the reliability of counting particles, as well as the granulometry estimation. Moreover, no information on the particle shapes is collected. The latter could be achieved by using efficient techniques based on vision systems [26].

Within the ongoing new SECESTA-VIASAFE project [27], further research efforts are under development to integrate the network of monitoring nodes developed by the SECESTA project with innovative sensing methodologies for the detection of volcanic ash granulometry.

In the considered context, this work focuses on a strategy that aims to detect and analyze ash particles from digital pictures through a dedicated image processing methodology, with the aim of extracting the core characteristics of volcanic particles. As outlined in [28], the measurement system uses a moving plate, which collects the falling down ash particles for a well-defined amount of time and then moves under the vision system, which is positioned in a protected room.

As respect to the preliminary study already presented in [29], this work presents a thorough description of the developed methodology and its assessment strategy, the extension of results related to the assessment process for a wide range of working conditions, and the preliminary results obtained in the case of a real sample of volcanic ash.

The obtained results are of strategic importance to the assessment of the performance and limitations introduced by the image processing paradigm, which would seriously impact the design and the choice of the hardware part of the vision system.

For each recognized particle, the developed paradigm estimates the perimeter and the area as well as major and minor axes of the rectangle bounding the particle. The latter is strictly related to the ash sizes, thus opening the possibility of classifying each particle as belonging to a specific class of granulometry. Future efforts will then be dedicated to estimating further features extracted from the core characteristics, allowing us to classify ash particles into different classes of pre-defined shapes.

The main novel outcomes introduced by the proposed approach are as follows:

- The capability of the solution proposed (including the image processing approach, the measurement setup, and the sensor network) to provide experts with a continuous-time awareness of the ash fall-out phenomenon with a high degree of spatial resolution. This is a mandatory information to feed models forecasting ash hazards and could fill the need for standard approaches for the measurement of volcanic ash granulometry.
- The idea of using a low-cost vision-based methodology to analyze volcanic ash, with particular regard to the possibility of gaining information about the dimensions of each detected particle. This aspect is very important considering the need for the development of wide sensor networks detecting ashes in large volcanic areas.
- The measurement protocol, including the image pre-processing and the estimation of core characteristics, which is novel with respect to the state of the art.
- The procedure that aims to assess the system performance against several influencing effects (such as particle position in the analyzed area, particle shape and rotation, particle color), which is fundamental in a real application scenario.

In the following, the image processing approach developed for particle detection and analysis and its assessment under different scenario is addressed. In particular, the next section provides an overview of the image processing paradigm. The assessments of the strategy proposed for the ash granulometry estimation is given in Section 3, while concluding remarks are given in Section 4.

## 2. The Proposed Approach

The focus of this paper is mainly related to an image processing methodology that is adopted to perform particle detection and analysis. Aspects related to the image acquisition system will not be developed in this work.

The proposed approach is based on the suitable processing of pictures of deposited ash grains, ideally on a white background, in order to maximize the contrast of the image. The rough image is processed through the application of a sequence of digital filters. The aim of the processing is to highlight each particle by initially converting all the pixels above a given brightness threshold into black pixels. The algorithm is then able to identify all the different particles in the picture and to estimate their characteristics (in pixels), such as the area and the perimeter. Given a binary image, a particle can be defined as a group of contiguous nonzero pixels in the image.

It must be highlighted that the conversion factor between pixels and the real object’s dimensions depends on the characteristics of the image acquisition system adopted and will not be addressed in this paper.

**Figure 1:**
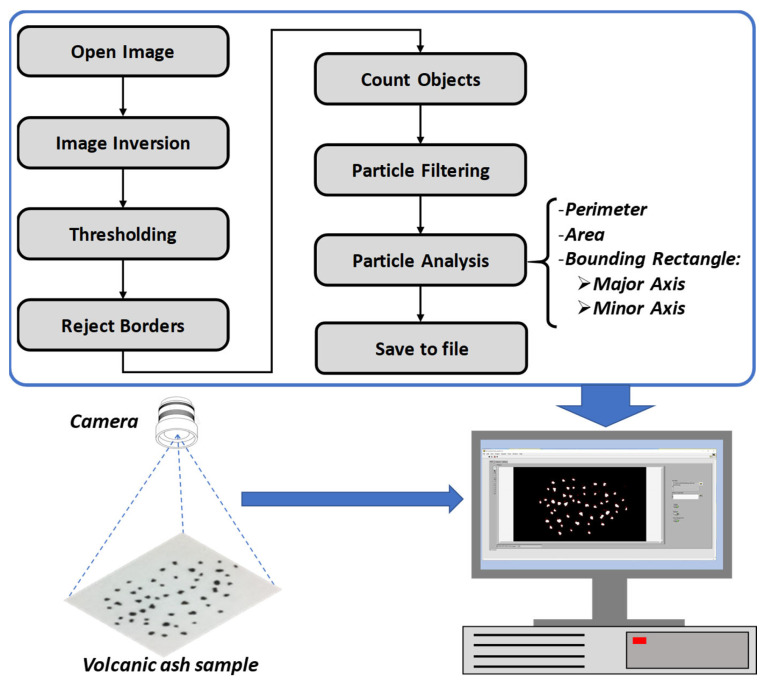
The image processing paradigm.

The detailed sequence of operation of the image processing algorithm is described by the flow chart shown in Figure 1:

- “Open image”: The stored image is opened and the image object is created.
- “Image inversion” inverts the pixel intensities of the image to compute the negative image.
- “Thresholding” converts the negative grayscale image to a binary image (each pixel can only assume the values “0”—black, or “1”—white) by comparing each pixel intensity of the image with a given threshold and assigning those pixels above the threshold to “white”, while the others are assigned to “black”.
- “Reject borders” eliminates particles touching the border of the analyzed image.
- “Count objects” locates and counts objects in the rectangular search area. This block uses a threshold on the pixel intensities to segment the objects from their background.
- “Particle filtering” filters out particles with a perimeter below a pre-defined threshold in order to reduce particle misidentification.
- “Particle analysis” returns the number of particles detected in the binary image and the characteristics of each detected particle; e.g., perimeter, area, bounding rectangle width and height.

In the following, the definitions of the main core characteristics of each particle are given:

- Particle perimeter (P): Length of a boundary of a region. Boundary points are the pixel corners that form the boundary of the particle.
- Particle area (S): Area of the particle.
- Bounding Rectangle: The width and height of the smallest rectangle bounding a particle.

The image processing paradigm sketched in Figure 1 was implemented in LabVIEWTM.

**Figure 2:**
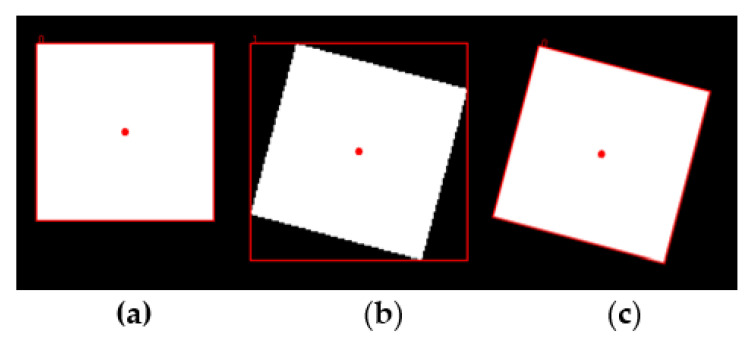
(**a**) Original sample; (**b**) rotated sample and the bounding rectangles estimated by the IMAQ Particle Analysis tool; (**c**) rotated sample and the bounding rectangles estimated by the Python script.

Since the IMAQ Particle Analysis tool defines the bounding rectangle with the sides parallel to the x and y axes, the results in the case of a rotated sample will introduce a considerable error, as in the case shown in Figure 2b. To overcome this problem, a dedicated Python script was implemented and integrated in the LabVIEW tool, which estimates the rotated bounding rectangle of a given particle, as shown in Figure 2c.

**Figure 3:**
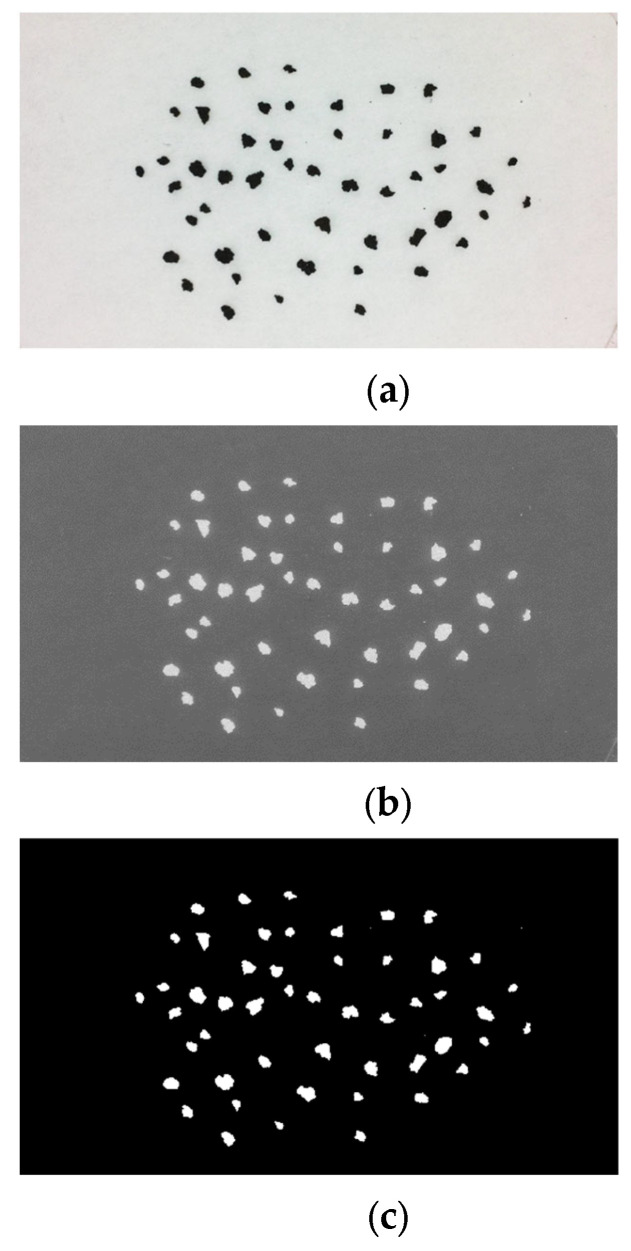
Overview of the image processing flow on a sample image of real volcanic ash particles (**a**); the image after the inversion (**b**) and thresholding (**c**) steps.

Figure 3 shows the results provided by the most relevant processing steps shown in Figure 1, in the case of an image with real samples of volcanic particles.

Actually, in order to reduce the possibility of particles overlapping, which may affect the performances of the image processing tool, the system includes a solution to shake the plate used to collect particles.

## 3. Assessment of the Image Processing Paradigm

**Figure 4:**
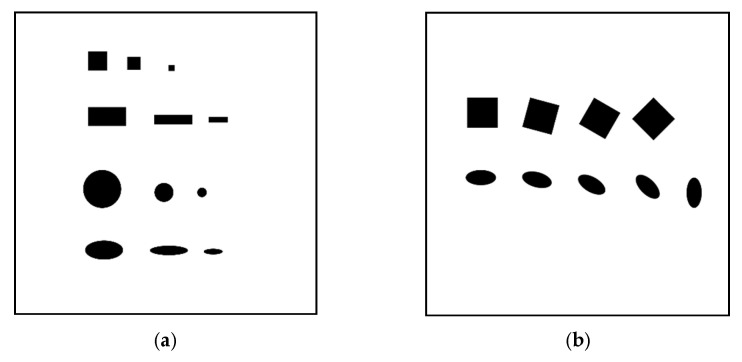
Two examples of test images used for the assessment of the image processing paradigm: (**a**) reference picture and (**b**) rotated picture.

The image processing paradigm was assessed by providing the LabVIEW tool with reference digital images, made of objects of known dimensions (in pixels), and analyzing the features estimated by the algorithm. Two examples of test images used during the assessment phase are shown in Figure 4a,b, showing non-rotated and rotated samples, respectively.

The nominal dimensions, in pixels, of the figures shown in two test images are reported in Table 1 and Table 2, respectively (figures are numbered from top-left to bottom-right).

The residuals between nominal and estimated values of the area, *S,* perimeter, *P,* major axis, *Amax*, and minor axis, *Amin*, of samples in the test images were used to assess the performance of the developed algorithm:

(1) $$J_{P}=100\cdot \frac{P_{n}-P_{e}}{P_{n}}$$

(2) $$J_{S}=100\cdot \frac{S_{n}-S_{e}}{S_{n}}$$

(3) $$J_{Amax}=100\cdot \frac{Amax_{n}-Amax_{e}}{Amax_{n}}$$

(4) $$J_{Amin}=100\cdot \frac{Amin_{n}-Amin_{e}}{Amin_{n}}$$

where *J_P_*, *J_S_*, *J_Amax_*, and *J_Amin_* represent the residuals for the perimeter, the area, and the major and minor axes, respectively. The subscript “*n*” indicates the nominal quantities, while the subscript “*e*” indicates the estimated quantities.

In order to assess the overall behavior of the processing paradigm, two synthetic indexes, *J_AV_* and *J_STD_*, were defined, which represent the mean value and standard deviation of each of the above-defined performance Indexes (1)–(4), estimated through the whole set of test particles:

(5) $$J_{AV,\;\;q}=\frac{1}{N}\sum_{i=1}^{N}J_{q,i}$$

(6) $$J_{STD,\;q}=\sqrt{\frac{1}{N-1}\sum_{i=1}^{N}{\left(J_{q,i}-J_{AV,q}\right)}^{2}}$$

where q={P, S, Amax,Amin} and *N* is the number of test particles identified in the image.

**Figure 5:**
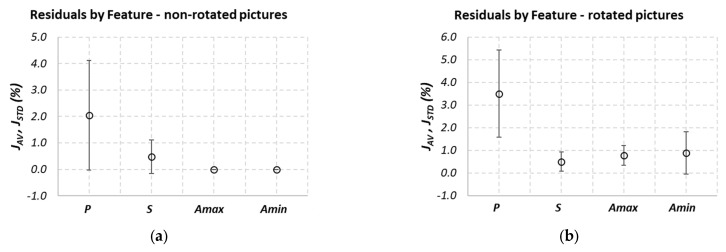
Results obtained for the test images, in terms of mean values (Index (5)) and standard deviations (Index (6)) of the performance Indexes (1)–(4), estimated for each particle core characteristic for the whole set of test particles: (**a**) reference image, (**b**) rotated image.

**Figure 6:**
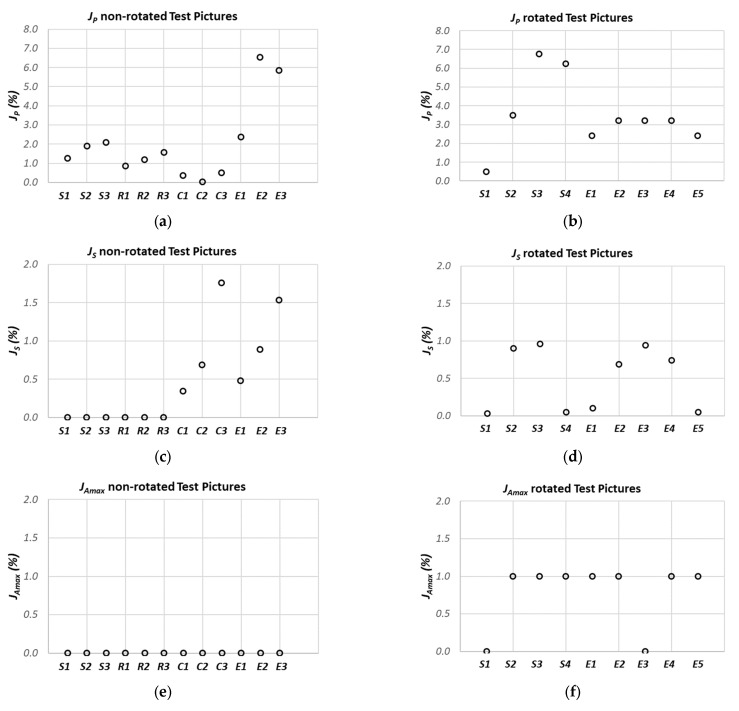

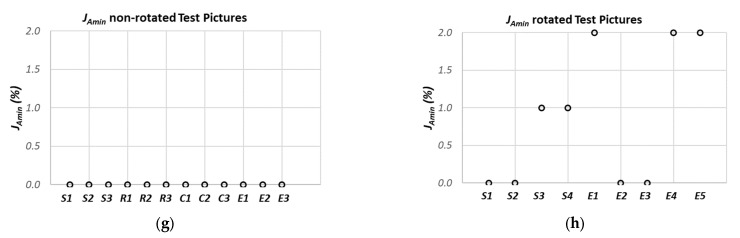
Behavior of Indexes (1)–(4) as a function of the test particles: (**a**,**c**,**e**,**g**) reference image, (**b**,**d**,**f**,**h**) rotated image. Particle legend: *S* = square, *R* = rectangle, *C* = circle, *E* = ellipse.

The behaviors of Indexes (5) and (6) are shown in Figure 5a,b for the non-rotated and rotated test pictures, respectively. The achieved results show that the most suitable feature in term of reliability is the estimated area, as it can be observed from the results shown in Figure 6a,b, for the reference and the rotated pictures, respectively.

Figure 6 show the behaviors of Indexes (1)–(4) as a function of the test particles: (a, c, e, g) show the non-rotated reference image, while (b, d, f, h) show the rotated image. The worst case, evidenced by the *J_S_* index, was obtained for the sample “Circle 3” in Table 1, which presents a residual between the nominal and estimated area equal to 1.76%.

The behaviors of Indexes (1)–(6), shown in Figure 5 and Figure 6, demonstrate the system’s robustness against the following factors:

- Particle shapes: The paradigm performances are not strictly influenced by the particle geometry (*S*, *R*, *C*, *E*).
- Particle dimensions: The paradigm performances are not strictly influenced by the particle sizes, both in the case of non-rotated pictures (*Si*, *Ri*, *Ci*, *Ei*, *i* = 1–3) and rotated pictures (*Si*, *Ei*, *i* = 1–4).
- Particle position: the accuracy of the particle core characteristic estimation is independent of the position of the sample in the inspected area.

Moreover, it can be affirmed that similar performances are obtained also in the case of rotated particles.

The robustness of the image processing algorithm was also tested against the particle colors, which, in real cases, may vary slightly as a function of the threshold value adopted to convert the grayscale image into the binary image. In particular, five set of reference pictures with different RGB combinations were used for the tests: from the darkest to the lightest, “Black” (RGB 0, 0, 0), “Grey 1” (RGB 65, 65, 65), “Brown 1” (RGB 84, 70, 51), “Grey 2” (RGB 130, 130, 130), and “Brown 2” (160, 82, 42). Threshold values were investigated in the range 160 ÷ 205, being 255 the full-scale.

**Figure 7:**
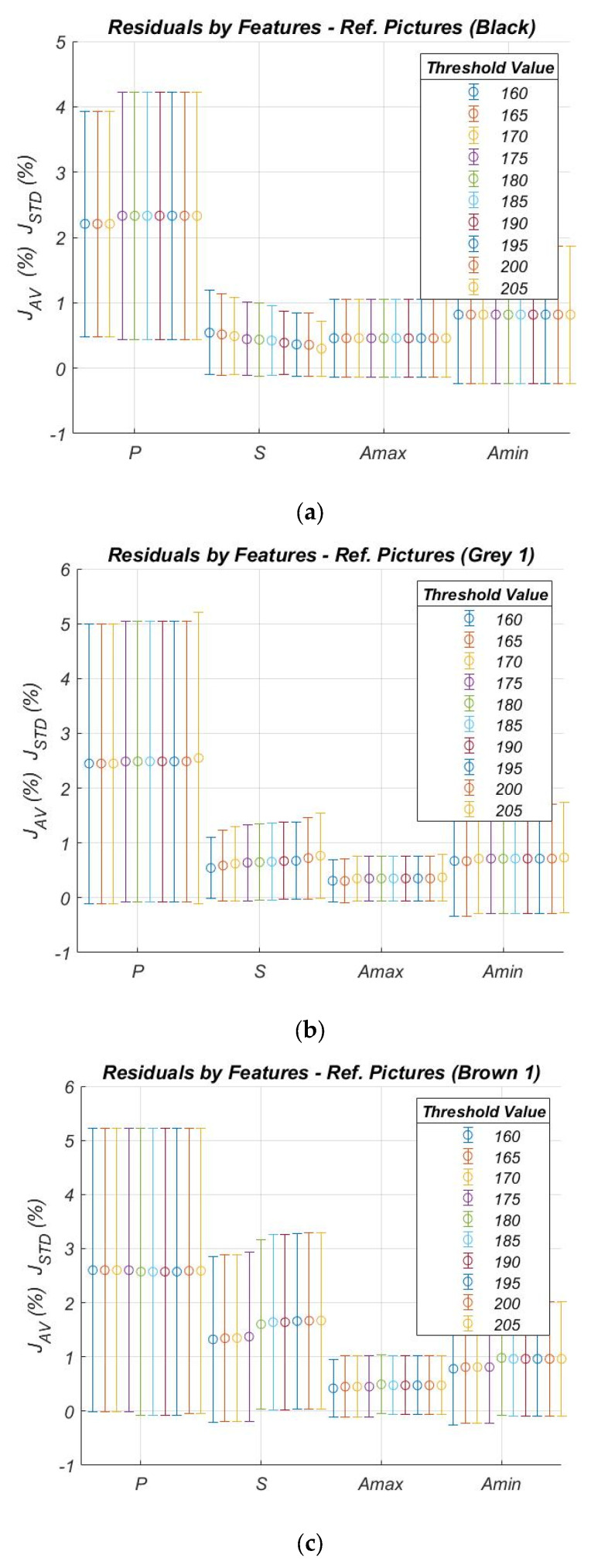
Results obtained for the reference picture “Brown 1”, in terms of mean values (Index (5)) and standard deviations (index (6)) of the performance Indexes (1)–(4), estimated for each particle core characteristic. (**a**) Black, (**b**) Grey 1, (**c**) Brown 1.

As an example, results obtained for Indexes (5) and (6), for three sets of reference pictures—Black, Grey 1, and Brown 1—are shown in Figure 7.

These results confirm the robustness of the adopted methodology against the color of ash particles. Moreover, it can be observed that the sensitivity against the threshold settings, in the considered range, is fairly low.

**Figure 8:**
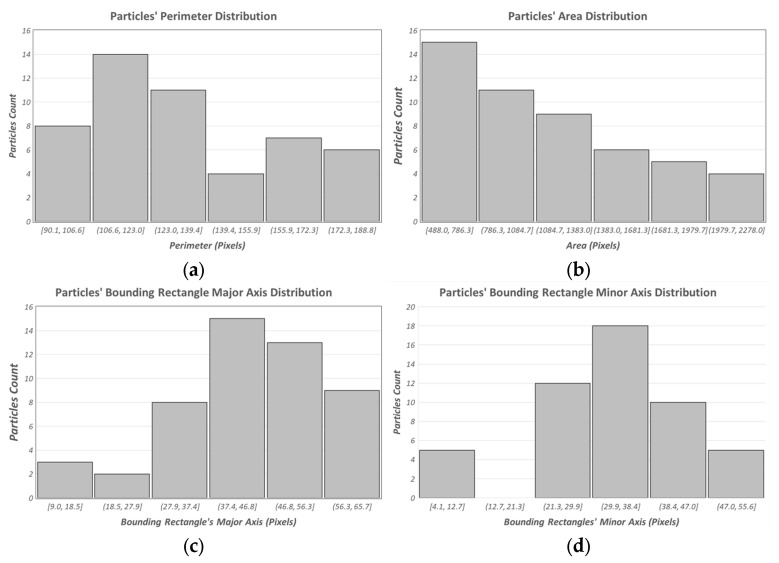
Distributions of core characteristics estimated for the case of particle samples shown in Figure 3a. (**a**) Perimeter, (**b**) area, (**c**) minor axis of the bounding rectangle, (**d**) major axis of the bounding rectangle.

As a preliminary result demonstrating the behavior of the tool developed in the case of real particle detection, Figure 8 summarizes the distributions of the core characteristics estimated for the case of the particle samples shown in Figure 3a. This kind of outcome is valuable to feed classification algorithms that aim to perform a suitable classification of volcanic ashes and to characterize the fall-out phenomenon.

## 4. Conclusions

In this paper, a vision-based approach for the estimation of characteristic parameters of volcanic ash particles has been presented. The tool developed works on pictures of deposited ash grains in order to estimate the main geometric features of all particles, such as the major and minor axes of the bounding rectangle, the perimeter, and the area. The proposed image processing strategy was implemented in LabView^TM^ and assessed by means of reference digital images resembling different operating condition in terms of particle size, shape, position in the inspected area, rotation, and color with respect to the reference background. The performance of the developed algorithm, estimated by considering the residuals between nominal and estimated values of samples’ features in the test images, confirms the suitability of the adopted approach.

In conclusion, considering that systems forecasting ash hazards require reliable systems that are able to detect the geometry of volcanic particles in real-time, and taking into account the absence of standard methodologies for granulometry measurement, it can be affirmed that the main outcome of this is related to the novel approach, exploiting the data provided by cameras to determine the main features of ash particles in real-time. Moreover, with the aim of developing a wide network of measuring nodes, the same algorithm was also converted into Python/Open CV in order to be compliant with low-cost embedded architectures implementing also the image capturing feature [28].

Future efforts will be dedicated to assessing the performances of the proposed image processing strategy implemented in a low-cost embedded architecture. In particular, the potential effect of the finite number of pixels of the vision system on the estimation of the particle morphology will be investigated. Actually, in the final system, including the camera and the processing unit, the pixel size will be fixed in order to be negligible with respect to the resolution expected by the system for estimating ash particle dimensions.

Moreover, starting from the above-mentioned core characteristics extracted for each particle, other features could be estimated that could be useful to feed classification paradigms that aim to split the captured particles into groups with a different granulometry and to assess their similarity to reference shapes. Another peculiar aspect worthy of further investigation would be the use of a stereo-vision system to reconstruct the 3D view of each particle.

## Figures and Tables

**Table 1:** Nominal characteristics, in pixels, of the test figures reported on the reference picture (from top-left to bottom-right).

	Nominal Geometrical Quantities (Pixels)			
Ref. Shapes	Perimeter	Area	Major Axis	Minor Axis
Square 1	400	10,000	100	100
Square 2	280	4900	70	70
Square 3	120	900	30	30
Rectangle 1	600	20,000	200	100
Rectangle 2	500	10,000	200	50
Rectangle 3	260	300	100	30
Circle 1	628	31,400	200	200
Circle 2	314	7850	100	100
Circle 3	157	1963	50	50
Ellipse 1	497	15,708	200	100
Ellipse 2	458	7854	200	50
Ellipse 3	232	2356	100	30

**Table 2:** Nominal characteristics, in pixels, of the test figures reported on the rotated picture (from top-left to bottom-right).

	Nominal Geometrical Quantities (Pixels)			
Ref. Shapes	Perimeter	Area	Major Axis	Minor Axis
Square 1	400	10,000	100	100
Square 2	400	10,000	100	100
Square 3	400	10,000	100	100
Square 4	400	10,000	100	100
Ellipse 1	248	3927	100	50
Ellipse 2	248	3927	100	50
Ellipse 3	248	3927	100	50
Ellipse 4	248	3927	100	50
Ellipse 5	248	3927	100	50

## Data Availability

Not applicable.
